# Tubulin's response to external electric fields by molecular dynamics simulations

**DOI:** 10.1371/journal.pone.0202141

**Published:** 2018-09-19

**Authors:** Joshua J. Timmons, Jordane Preto, Jack A. Tuszynski, Eric T. Wong

**Affiliations:** 1 Brain Tumor Center & Neuro-Oncology Unit, Beth Israel Deaconess Medical Center, Harvard Medical School, Boston, Massachusetts, United States of America; 2 Department of Physics, University of Alberta, Edmonton, Canada; 3 Department of Oncology, University of Alberta, Edmonton, Canada; 4 Department of Mechanical and Aerospace Engineering, Politecnico di Torino, Corso Duca degli Abruzzi, Torino, Italy; Institut de Genetique et Developpement de Rennes, FRANCE

## Abstract

Tubulin heterodimers are the building blocks of microtubules and disruption of their dynamics is exploited in the treatment of cancer. Electric fields at certain frequencies and magnitudes are believed to do the same. Here, the tubulin dimer’s response to external electric fields was determined by atomistic simulation. External fields from 50 to 750 kV/cm, applied for 10 ns, caused significant conformational rearrangements that were dependent upon the field’s directionality. Charged and flexible regions, including the α:H1-B2 loop, β:M-loop, and C-termini, were susceptible. Closer inspection of the α:H1-B2 loop in lower strength fields revealed that these effects were consistent and proportional to field strength, and the findings indicate that external electric fields modulate the stability of microtubules through conformational changes to key loops involved in lateral contacts. We also find evidence that tubulin’s curvature and elongation are affected, and external electric fields may bias tubulin towards depolymerization.

## Introduction

α- and β- tubulin heterodimers spontaneously assemble end to end to form protofilaments, and the helical arrangement of 13 protofilaments constitutes microtubules that are central to cellular rigidity, division, motility, and trafficking of intracellular proteins [1–4]. As the driving force for sister chromatid segregation in mitosis, microtubules have long been targeted by chemotherapies using pharmacological agents that stabilize MTs such as paclitaxel, and those that destabilize them such as vincristine, and vinblastine [5,6]. More recently, microtubules have become the target of a novel treatment modality: electric fields. Alternating electric fields at 2.5 V/cm with a frequency of 100–300 kHz, known as Tumor Treating Fields (TTFields), disrupt microtubules *in vitro*. While no definitive mechanistic model of their action has been established, a putative explanation claims that this is due to the microtubules’ large intrinsic charge and dipole [7–10] which interacts with electric fields and their gradients. In a major development in the area of cancer therapy, Optune®, a device for administering the fields to patients, was recently approved by the FDA to treat glioblastoma [11–13].

Compared to TTFields, external electric fields (EEFs) of much greater strengths but shorter timescales can also eliminate cancer cells *in vitro* and *in vivo*. Nanosecond pulsed electric fields (nsPEFs)—static EEFs of tens of kV/cm that are applied repeatedly over nanosecond durations [14,15]—have demonstrated anti-cancer potential against Jurkat and HL-60 cells *in vitro* at 300 kV/cm [15], papillomas and squamous cell carcinoma *in vivo* at 40 kV/cm [16], and many other cancer models [17–23]. Several mechanisms have been proposed to explain nsPEFs’ effects, but their creation of nanopores, which allow an influx of Ca^2+^ from extracellular and intracellular sources, and reduction of mitochondrial membrane potential have received the most attention [14,24,25].

More recent investigations have demonstrated that nsPEFs affect the cytoskeleton. Nanosecond pulsed electric fields cause a breakdown of actin filaments with concomitant cell rounding [26–29] and 44 kV/cm pulses induce microtubule clearance in U87 human glioblastoma cells within minutes, all without observable Ca^2+^ influx and/or osmotic swelling [30]. These findings of microtubule breakdown [30], in conjunction with those of Kirson et al. [31] in TTFields, suggest that EEFs may destabilize microtubules directly in addition to various indirect effects that have been proposed [8].

**Fig 1:**
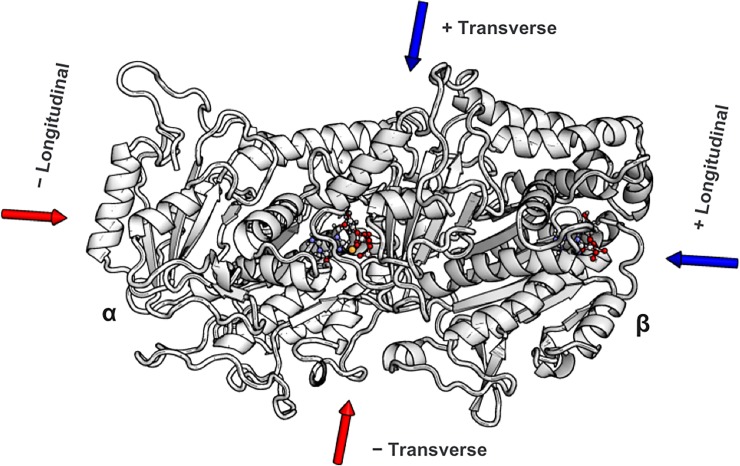
Directionality of the applied electric fields. Electric fields were applied along the direction of the dipole (transverse axis) and along the vector between the beta and alpha monomers’ center of mass (longitudinal axis), both in reference to the equilibrated dimer’s initial position.

Other investigations of tubulin in EEFs have found that microtubules can be aligned with an applied electric field [32,33] and that EEFs reduce the Young’s modulus of a tubulin heterodimer [34]. Despite these earlier findings, the precise atomic-level details of a single dimer’s response to EEFs are unknown. Therefore, we performed an investigation of these effects through Molecular Dynamics, applying EEFs along multiple directions (Fig 1) at time scales and field strengths consistent with nsPEFs (36–38), the results of which are important for understanding macroscopic observations like nsPEF-induced depolymerization. Notably, the α:H1-B2 loop and β:M-loop, that are integral to lateral contacts between protofilaments, are especially susceptible to the influence of electric fields, and the bend angle and elongation of tubulin are affected, which may accelerate the depolymerization process.

## Results and discussion

### Tubulin’s structure and dynamics are affected by EEFs

#### Structural change as measured by root mean standard deviation

**Fig 2:**
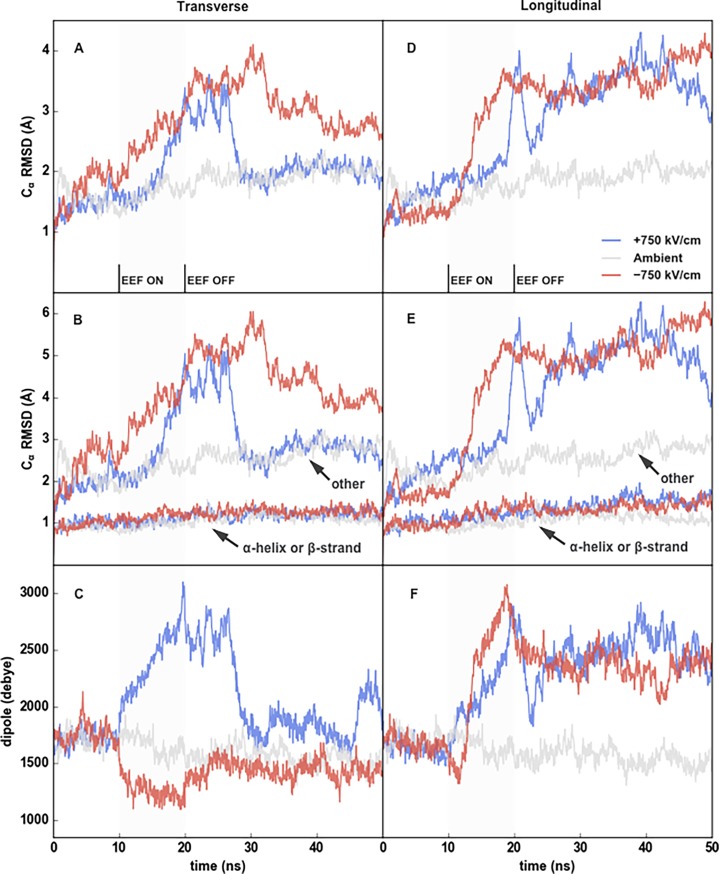
Structural rearrangement in the presence of a strong EEF. Cα RMSD of a tubulin dimer in the transverse (a) and longitudinal (d) directions. Cα RMSD again but with delineation of displacement in alpha helices and beta sheets versus other structures in the transverse (b) and longitudinal (e) directions. Total dimer dipole magnitudes for the transverse (c) and longitudinal (f) directions.

We first investigated large-scale EEF-induced changes to tubulin’s root mean standard deviation (RMSD) and dipole magnitude. By both measurements, a 750 kV/cm EEF caused significant structural changes in all directions tested (Fig 2). Most of the change in RMSD was in loops and turns. As expected, we found that rigid structures like alpha helices and beta sheets remained relatively fixed, with an RMSD for alpha carbons close to 1 Å, whereas other structural motifs were more sensitive to the EEFs and exhibited an RMSD of 5–6 Å. By comparison, the same flexible motifs showed an RMSD of around 2–3 Å in the dimer unexposed to an EEF. These more-flexible residues were classified as, in order of prevalence, coils, hydrogen bonded turns, and 3_10_ helices (Fig 2B and Fig 2E). The relaxation time was less than 30 ns, when the EEF was applied along the dimer’s transverse axis (Fig 2A), but lasted longer when applied along the longitudinal axis (Fig 2D). Microtubules clearance is observable in nsPEFs applied at 10 Hz, or with 100 ms between their application, which means that there is likely sufficient time for total relaxation in a single dimer between pulses.

#### Increase of dipole moment by EEFs

In all but the negative transverse direction, or opposite the dimer’s dipole, the EEF doubled the magnitude of tubulin’s dipole moment (Fig 2C and Fig 2F). Increases in dipoles were longer lasting when the EEF was applied along the longitudinal versus transverse axis, a finding similar to our results in RMSD (Fig 2C and Fig 2F). This effect is explainable by coupling of charged residues with the EEF, in which the dimers’ natural charge distributions were exaggerated (in all but the negative transverse simulation). Atomistic simulations of insulin and lysozyme in EEFs have demonstrated that protein dipoles are affected by the localized rearrangement of charged sidechains and loops [35–37]. Additionally, Ojeda-May et al [38] and Budi et al [36] found that EEFs can convert beta sheets to alpha helices or destabilize alpha helices, respectively, but we observed neither in the 750 kV/cm EEFs studied here (S1 Fig).

### Rearrangement is greatest in the α:H1-B2 Loop, β:H6-H7 Loop, and C-termini

#### Charged, flexible regions undergo large per-residue displacement

**Fig 3:**
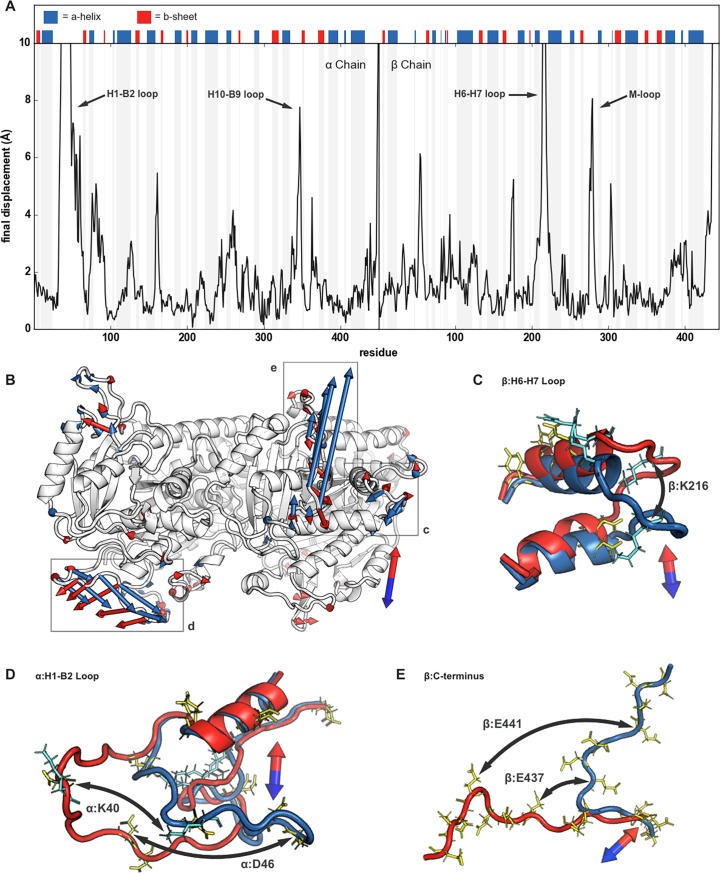
Displacement induced by transverse electric fields. (A) Displacement distance (Å) of Cα atoms between final tubulin structures after 10 ns of 750 kV/cm in the positive and negative transverse directions (after superimposition of structures). (B) Displacements exceeding 2 angstroms are visualized by vectors from the residues in the initial, equilibrated structure (white) to the final structure after application along the negative direction (red arrows) and the positive direction (blue arrows). For clarity, every other displacement vector is shown. (C, D, E) Final positions of the dimer are visualized after 750 kV/cm in the negative (red) and positive (blue) transverse directions for the β:H6-H7 Loop, α:H1-B2 Loop, and β:C-terminus. Positively and negatively charged residue side chains are represented by light-blue and yellow sticks, respectively.

**Fig 4:**
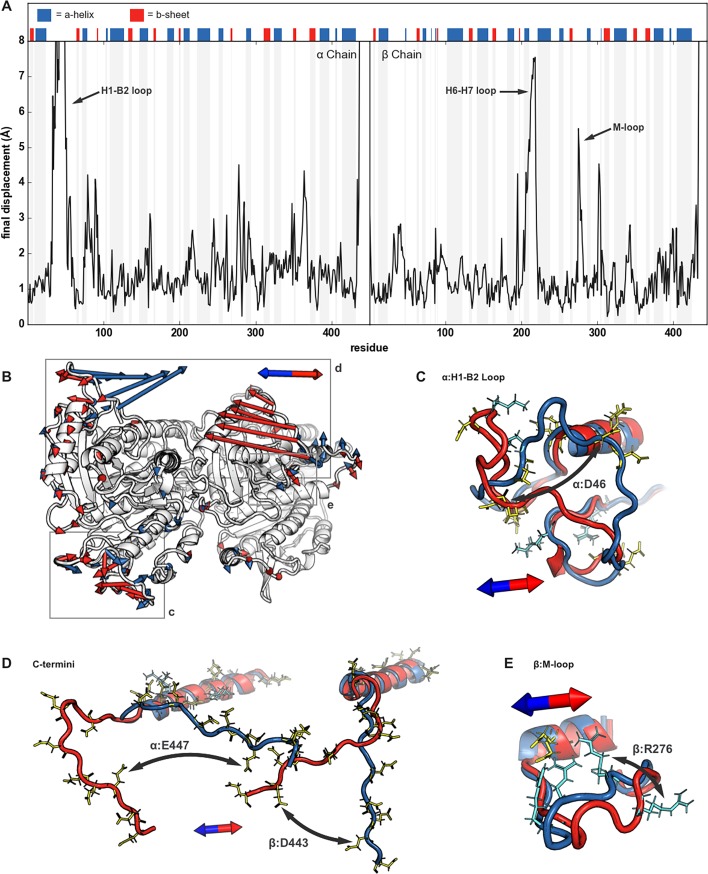
Displacement induced by longitudinal electric fields. (A) Analogous to Fig 3, but comparing displacement between structures after application of longitudinal EEFs. (B) Displacement vectors are shown from every other Cα, for clarity. (C, D, E) Final positions are visualized for the heterodimer after 750 kV/cm in the negative (red) and positive (blue) longitudinal directions for the α:H1-B2 Loop, C-termini, and M-loop.

To gain a better understanding of EEFs’ effects on the tubulin dimer, we superimposed final structures, after 10 ns of EEF exposure in opposite directions, and calculated displacement on a residue-by-residue basis. In flexible regions, structural rearrangement was dependent upon the residue’s root mean square fluctuation (RMSF) (S2 Fig) and charge; positive and negative residues move towards the EEF’s anode and cathode, respectively. There was overlap in the affected regions within dimers in the transverse and longitudinal EEFs. After exposure to EEFs along the dimer’s transverse axis (Fig 3B), displacement was greatest in the α:H1-B2 loop (Fig 3D), α:H4-B5 loop, β:H6-H7 loop (Fig 3C), β:M-loop, and C-termini of both monomers (Fig 3E). Similarly, the longitudinal EEFs greatly affected the α:H1-B2 loop (Fig 4C), β:H6-H7 loop, β:M-loop (Fig 4E), and both C-termini (Fig 4D).

C-termini exposed to EEFs showed large movements relative to those that were unexposed, but the extent of their rearrangement was dependent on the field’s direction. While the C-termini of the α-monomer and β-monomer shifted inwards in the positive and negative longitudinal EEF, respectively, neither were significantly displaced when the EEF was applied along the opposite, outward direction (Fig 4B). This, plus other research indicating that there are frequent interactions between each monomer’s C-terminus and the other’s H11 [39], suggests that the C-termini take an “inward” trajectory from a position on the surface of the dimer. In opposition to this movement is the attraction between the negative C-termini and the dimer’s positively charged surface. Our equilibrated structure contained salt bridges from α:311K to α:447E and β:292K to β:445E (S3 Fig); the C-termini would necessarily need to escape their attraction to the dimer’s positive surface along an inward low-energy path and the EEFs’ direction influences whether they reach such an extended state.

EEF-induced modulation of C-termini would have biological relevance because of effects on the dynamics of kinesin [40], dynesin [41], and other microtubule-associated proteins, all of which directly interact with the C-termini of microtubules. In fact, post-translational modifications and removal of the C-termini have already been implicated in changes to motor protein velocity and processivity [41,42]. The interaction between the C-termini and EEFs may be further complicated by post-translational modifications. One such modification is polyglutamylation, which is common to cancer cells, and would result in the addition of negatively charged glutamate amino acids to the already negatively charged C-termini [43] making the latter protrude even more from the mostly negatively charged surface of microtubules. Further investigations of the C-termini’s response to EEFs are needed to characterize their transition between contracted and extended states [44] and whether such changes affect the dynamics of microtubule-associated proteins.

#### Per-residue fluctuations are augmented in EEFs

**Fig 5:**
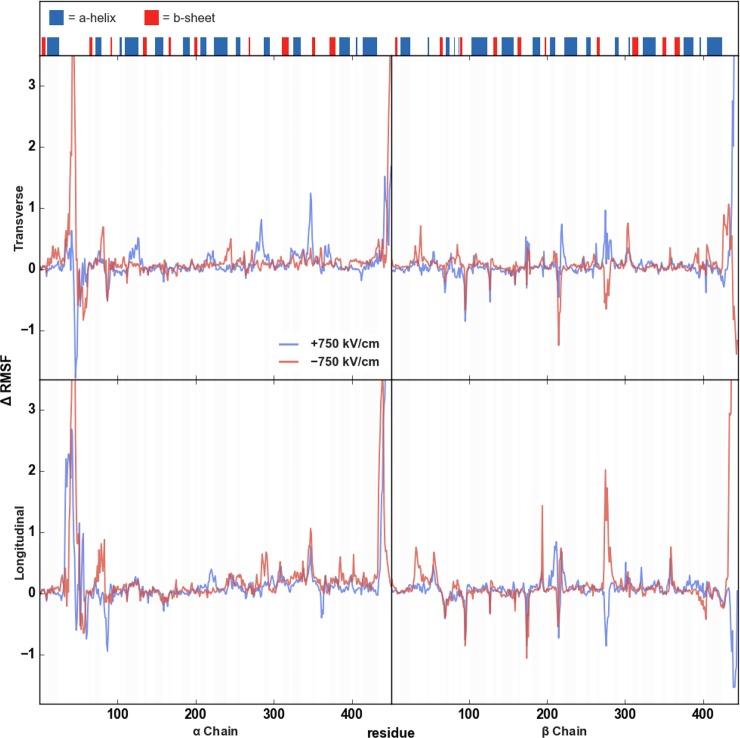
Change in per-residue RMSF. Changes in residue fluctuations for a tubulin dimer in a 750 kV/cm external electric field compared to a tubulin dimer not exposed to an external electric field (Δ*RMSF* = *RMSF*_*EEF*_ − *RMSF*_*AMB*_). The positive transverse (blue) and negative transverse Δ*RMSF* plots are along the top row, while the positive longitudinal (blue) and negative longitudinal (red) Δ*RMSF* plots are along the bottom.

We also investigated how EEFs affect residue fluctuations. We calculated change in RMSF as Δ*RMSF* = *RMSF*_*EEF*_ − *RMSF*_*AMB*_ where *RMSF*_*EEF*_ represents fluctuations of each residue in the dimer exposed to an EEF and *RMSF*_*AMB*_ denotes fluctuations in dimer unexposed to an EEF. Therefore, a residue with a large positive Δ*RMSF* experienced a significant increase in its fluctuation in an external electric field (relative to the same residue in the dimer unexposed to an external electric field); conversely, a residue with a large negative Δ*RMSF* was found to have stabilized by the field. The results (Fig 5 and S4 Fig) further demonstrate the interaction between EEFs and the α:H1-B2 loop, α:H10-B9 loop, M-loop, and C-termini. For these regions, the EEFs’ influence on |Δ*RMSF*| was consistent, regardless of the direction in which the EEF was applied, but whether Δ*RMSF* was positive or negative was variable. For example, there was an increase in fluctuation for the M-loop in both the positive transverse and, especially, negative longitudinal directions. But the same loop showed reduced fluctuation in the negative transverse and positive longitudinal directions. The directional dependence was most apparent in the α:H1-B2 loop which, for three of the four directions tested, experienced the greatest increase in fluctuation among all internal structures, yet was rigidified in the positive transverse EEF.

#### EEF perturbation results are consistent at lower magnitudes

**Fig 6:**
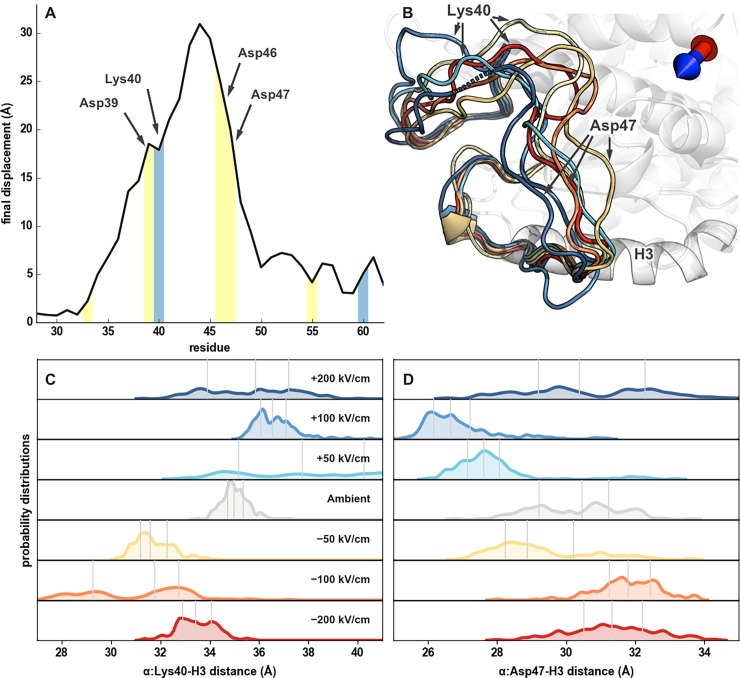
α:H1-B2 Loop’s response to transverse external electric fields. (A) Final per-residue displacement, after application a 750 kV/cm EEF along the transverse axis, between a dimer exposed to a positive and negative transverse direction. Negatively and positively charged residues are highlighted with yellow and light-blue, respectively. (B) Average positions of the α:H1-B2 loop, during the final 5 ns of EEF application, along the transverse direction. Loop colors of dark blue, blue, light blue, yellow, light orange, orange, and red correspond to +200, +100, +50, 0, -50, -100, and -200 kV/cm field strengths, respectively. The positive and negative EEF directions are indicated with a blue and red arrowhead, respectively. (C, D) Probability distributions for the distance between α:40K and α:H3 and between α:47D and α:H3, using snapshots from the final 5 ns of EEF exposure, correspond to outward movement of the first and second half of the α:H1-B2 loop, respectively. Quartiles for distance measurements are indicated with vertical gray lines.

Given its notable susceptibility to 750 kV/cm EEFs, measurable by both displacement and Δ*RMSF*, we examined the robustness of α:H1-B2 loop’s response to EEFs of lower strengths. We ran 10 ns simulations of EEFs applied along the positive and negative transverse directions at 50, 100, and 200 kV/cm and then visualized the average positions of the loop over the last 5 ns (Fig 6B). We also characterized the loop’s movement using measurements of two charged amino acids—greater distances between α:40K and α:H3 (Fig 6C) and α:47D and α:H3 (Fig 6D) correspond to outward movements of the first and second half of the α:H1-B2 loop, respectively. Inspection of the α:H1-B2 loop’s response to lower strength EEFs revealed a change in conformation that was proportional to field strength. The greatest effect was observed in the movements of α:40K.

As a positively charged residue, α:40K was more frequently in an “outward” conformation in a positive transversal field and vice versa, even at 50 kV/cm (Fig 6C). Conversely, the negatively charged α:Asp47 was more likely to be in an “inward,” closer to the dimer conformation in a positive transverse EEF. Given the field magnitudes and exposure duration tested here, the results indicate that nsPEFs induce rearrangement in the α:H1-B2 loop. The findings are relevant to a mechanistic explanation of the breakdown of microtubules under the influence of EEFs.

A tubulin dimer in a microtubule is held in place through loop-loop interactions. A dimer within a microtubule has both longitudinal and lateral contacts with its own and adjacent protofilaments, respectively [4,45,46]. In the energetically favorable B-lattice confirmation, lateral contacts between the α monomers of two protofilaments include hydrogen bonds between the H1-B2 loop on one side and the M-loop on the other [47,48]. Similarly, lateral contacts between β monomers include hydrogen bonds between the M-loop of one dimer and the H1-B2 loop of the other [2,4,47]. Given that these regions are susceptible to EEFs, we conjecture that this induced rearrangement may promote microtubule catastrophe.

### Tubulin’s essential dynamics are affected by EEFs

**Fig 7:**
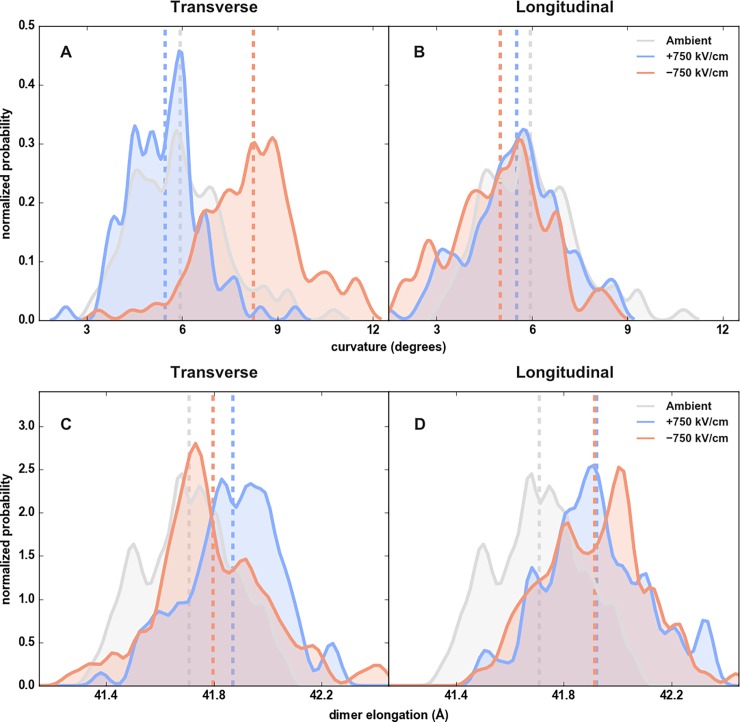
Tubulin essential dynamics in an external electric field. (A, B) Normalized probability distributions of tubulin’s curvature and dimer elongation for the last 5 nanoseconds of EEF application. Curvature was calculated by the method presented in [51]. Elongation was measured as the distance between the center of mass of each monomer’s alpha helices. Mean curvature and compaction are shown by dashed lines.

Beyond the per-residue effects of EEFs, we asked whether the lower-frequency motions of tubulin were also affected. Specifically, intradimer curvature and elongation are two modes that are biologically relevant for the polymerization and depolymerization of microtubules [49,50]. These two essential modes in microtubule dynamics were calculated over the last 5 ns of exposure to 750 kV/cm EEFs for all field directions (Fig 7). The negative transverse EEF promoted the greatest change in curvature, with the mean bend angle at 5.9° and 8.2° for the unexposed and negative transverse EEF, respectively (Fig 7B). Furthermore, EEFs in all four directions promoted elongation, though the greatest increases were in the longitudinal EEFs (Fig 7D).

The curvature of tubulin has long been linked to the dynamics of microtubules. The straight conformation is found in zinc-induced protofilament sheets [52] and are characteristic of stable microtubules [2], while the curved conformation is found in depolymerization peels, which are sometimes referred to in the literature as “ram’s horns” [4,53]. A recent model, based on structures produced with cryo-electron microscopy, proposed that the curved dimer conformation is an energetically preferred state that alleviates the strain of elongation produced during the depolymerization process [4]. Given our findings that EEFs can bias tubulin dimers towards bent (Fig 7B) and elongated conformations (Fig 7D), we posit that modulation of essential dynamics is another means by which nsPEFs affect microtubule stability.

While our model investigates the effects of EEFs on a single tubulin dimer, other groups have proposed that nsPEFs’ effects on the cytoskeleton are due to electrophoresis of the large, negatively charged microtubules [30]. In other words, microtubule breakdown in nsPEFs stems from macro-scale forces, because an unaligned microtubule experiences a lateral electrophoretic force in electric fields [54], and the breakdown of loop-loop interactions occurs secondarily. We did observe an increase in the mean squared displacements of dimers that were proportional to the EEFs’ magnitude (S5 Fig), but insight into large-scale, electrophoretic breakdown of microtubules would require either a macroscopic model of microtubule networks or an *in vitro* experiment on microtubule buckling in a range of external electric fields. Relatedly, buckling during the alignment process appears to be modulated by loop-loop interactions between adjacent protofilaments, as evidenced by microtubule response to acetylation of α:40K [55]—so EEF-induced changes in loop-orientation may affect microtubules’ plasticity and resistance to buckling at the level of the dimer. Future experiments may further this work by increasing tubulin’s number—by simulating either a full or partial cross-section of a microtubule with lateral contacts [48]—or by investigating the effects of hydrolysis of GTP on the dimer’s response to EEFs.

## Conclusions

In this paper, we have reported on our computer simulations of the effects of externally applied alternative electric fields on the structure and dynamics of tubulin dimers. This was motivated by several experimental observations concluding that microtubules become unstable when exposed to sufficiently strong electric fields. In our simulations, we have observed displacement in the charged and flexible regions of tubulin, including the α:H1-B2 loop, β:M-loop, and C-termini. The changes to the α:H1-B2 loop were persistent at lower magnitude EEFs, and there were also detectable perturbations to tubulin’s curvature and elongation. Based on these observations, we propose a mechanistic explanation of the microtubule breakdown in EEFs, which is most likely to involve promotion of depolymerization through weakened lateral bonds and increased dimer strain. Finally, we have found significant effects of the field’s orientation on the conformational rearrangements of tubulin dimers, an observation that should encourage an experimental validation.

## Materials and methods

We created a 3D model of a tubulin heterodimer by modifying a crystal structure of tubulin bound to Taxol: PDB ID: 1JFF [56]. Preparing this structure for computer simulations, we removed Taxol, added missing residues α:1, α:35–60, α:440–451, β:1, and β:428–445, and energy minimized using Modeller [57].

Note that the residue indices referenced here are ungapped and therefore differ from those reported by Löwe et al [56]. The final structure included the dimer’s C-termini and consisted of 451 residues in α-tubulin plus GTP and one Mg^2+^ atom, both of which are bound to α-tubulin, and 445 residues in β-tubulin plus GDP, which is bound to β-tubulin. We included missing hydrogen atoms and delta protonated histidine residues with psfgen [58]. We then solvated the dimer in TI3P3 water atoms in a rectangular box with 20 Å of padding along each axis using VMD’s Solvate plugin. This box sizing was chosen with consideration of C-terimini movements to prevent interaction with the dimer’s image. To neutralize the dimer and produce a NaCl molarity of 150mM, we added 185 Chloride and 240 Sodium ions *via* VMD’s Autoionize plugin; ultimately, the periodic cell was 110 x 150 x 110 Å along the x, y, and z axes, respectively, and contained 164,493 water atoms.

Long-range electrostatics effects were treated with Particle-Mesh Ewald sum and a 12 Å cutoff [59]. We applied rigid bonds to hydrogen bonds, used a 2 femtosecond timestep, and saved frames to trajectories every 25 ps. We energy minimized the solvated structure for 25 ps, equilibrated it for 500 ps in an NVT ensemble, then equilibrated it for 1 nanosecond in an NPT ensemble. Because of 1JFF’s unfavorable initial conformation, we performed extensive additional energy minimization of the dimer by running it for an additional 100 ns in an NPT ensemble until convergence was definitive in RMSD. All simulations were run with the CHARMM36 force field [60]. Notably, CHARMM36 is a non-polarizable force field in which dipoles are measured purely by the distribution of fixed charges. In future studies, electronic polarization by the EEF and or other modalities could be accounted for with a polarizable force field like Drude-2013 or AMOEBA-2013 [61–63]. NAMD 2.10 [64] was used to run the simulations on clusters of 64 nodes, each containing 16 cores, connected by Infiniband in a BlueGene/Q cluster. We ran equilibration and production simulations at 310°K and coupled them to a Nosé-Hoover-Langevin piston for pressure control and used Langevin dynamics for temperature control.

We simulated static electric fields with the External Electric Field module of NAMD and applied them along four directions that we define as the positive and negative of the transverse and longitudinal axes, respectively (Fig 1). The positive transverse direction was that of the dimer’s dipole moment at the start of the production runs while the negative transverse direction was its negative. The positive longitudinal direction was along the vector formed from the beta to the alpha monomers’ center of mass at the beginning of the production runs; here again, the negative longitudinal vector was its negative. In addition to the control run, we ran simulations of EEFs at 750 kV/cm along each of the four major directions; we chose this initial magnitude based on other Molecular Dynamics studies of proteins in EEFs found in the literature [35,65,66]. We performed each production run for 50 ns and applied the EEFs between the 10 and 20 ns time points (using a 10 ns duration that is typical of nsPEFs [30]). Additionally, we simulated lower strength fields at 200, 100, and 50 kV/cm along the two transverse directions for 10 ns using the positions and velocities of the ambient production run at the ten-nanosecond mark for the first frame.

Analysis was performed with modules and scripting in VMD [58]. Per-residue displacement, referred to here as “final displacement,” was calculated as the distance between alpha carbons after superimposing heterodimers exposed to two EEFs of opposite directionality, which is loosely based on the concept developed by Hekstra et al [67]. Tubulin’s intradimer curvature (or bend angle) was calculated by the method described by Pecquer et al [51] and characterized by Peng et al [53]. Briefly, we calculated curvature as the intersection angle between the two best fit lines—created with the atoms of α:H7 and β:H7—from the Taxol bound tubulin dimer, PDB ID: 1JFF, and the dimer under study, after superimposing the two structures by the atoms of their α:H7s. We measured dimer elongation as the distance between the centers of mass from all alpha helices atoms in the α and β monomers, using atoms classified as belonging to alpha helices in the initial equilibrated structure.

## Supporting information

S1 Fig Secondary residue classifications in a 750 kV/cm EEF. (A, B) The total number of residues that are classified as alpha helices or beta sheets during the application of 750 kV/cm EEFs of four directions: positive transverse, negative transverse, positive longitudinal, and negative longitudinal.
(TIFF)

S2 Fig Final displacement in a transverse external electric field and RMSF. Final displacement, measured as the distance between residues after 10 ns exposure to a positive and negative transverse EEF (black). Cα RMSF per residue of a tubulin heterodimer, from the 10 to 20 ns time frame, without exposure to an EEF (blue).
(TIFF)

S3 Fig Salt bridges between the C-termini and the dimer surface. Representative salt bridges experienced by the C-termini when in a contracted state (lying along the dimer’s surface). A distance cutoff of 3.2 Å and the positions of the initial frame, after equilibration, were used to identify possible salt bridges.
(TIFF)

S4 Fig Visualization of change in per-residue RMSF. Same as Fig 4 but with ΔRMSF of each residue mapped onto the structure. Red and blue indicate an increase and decrease in RMSF, respectively. Field directions are indicated with arrows.
(TIFF)

S5 Fig Mean standard displacement of tubulin in a transverse external electric field. Mean standard displacement (Å^2^) of tubulin from the start to the end of the application of a transverse EEF.
(TIFF)
