# Supplementary figures and images for: Tubulin's response to external electric fields by molecular dynamics simulations
Source: PLoS One. 2018 Sep 19;13(9):e0202141. doi: 10.1371/journal.pone.0202141 (PMC6145594; doi:10.1371/journal.pone.0202141)

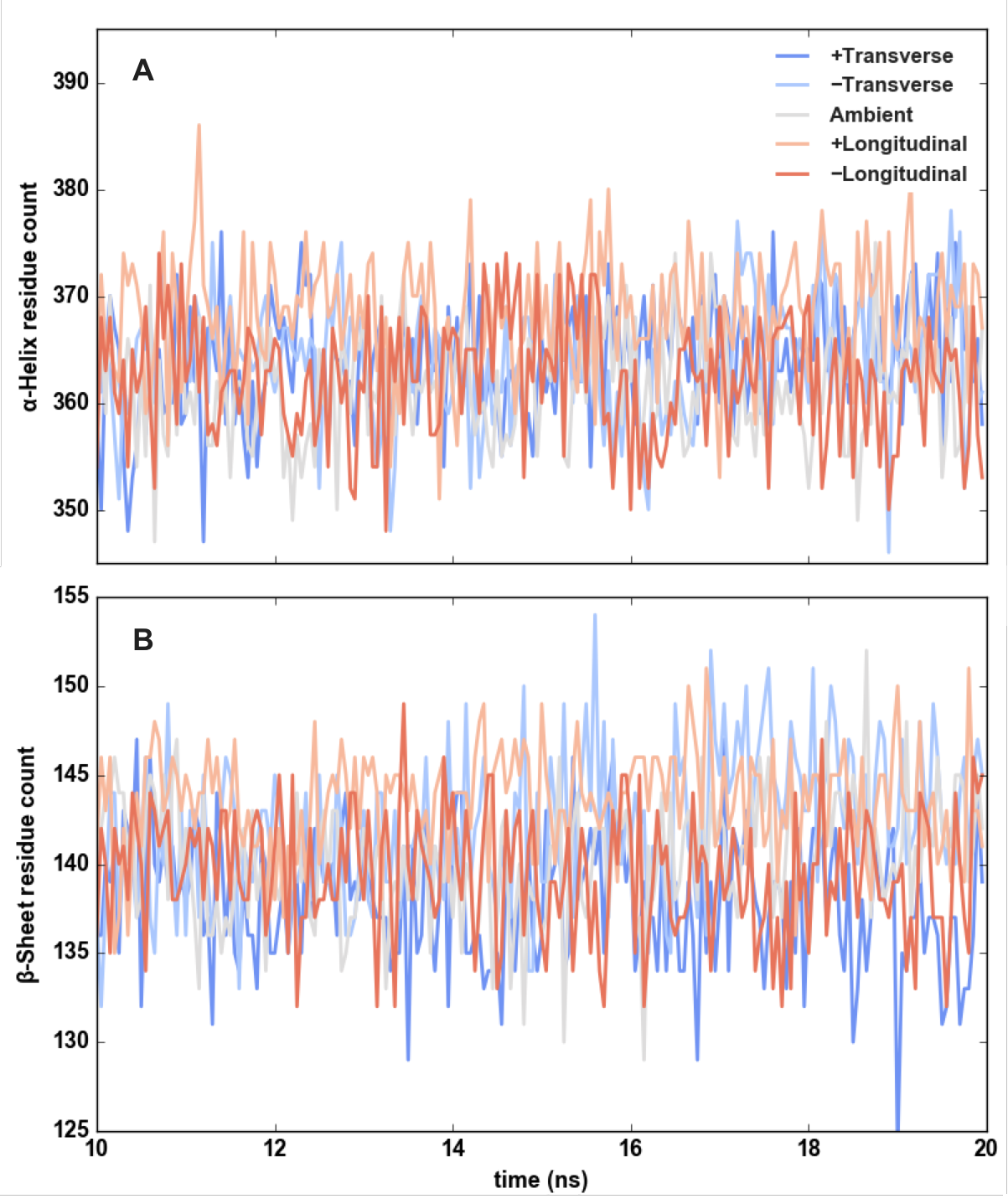

Supplement: S1 Fig — (A, B) The total number of residues that are classified as alpha helices or beta sheets during the application of 750 kV/cm EEFs of four directions: positive transverse, negative transverse, positive longitudinal, and negative longitudinal. (TIFF) [file pone.0202141.s001.tiff]

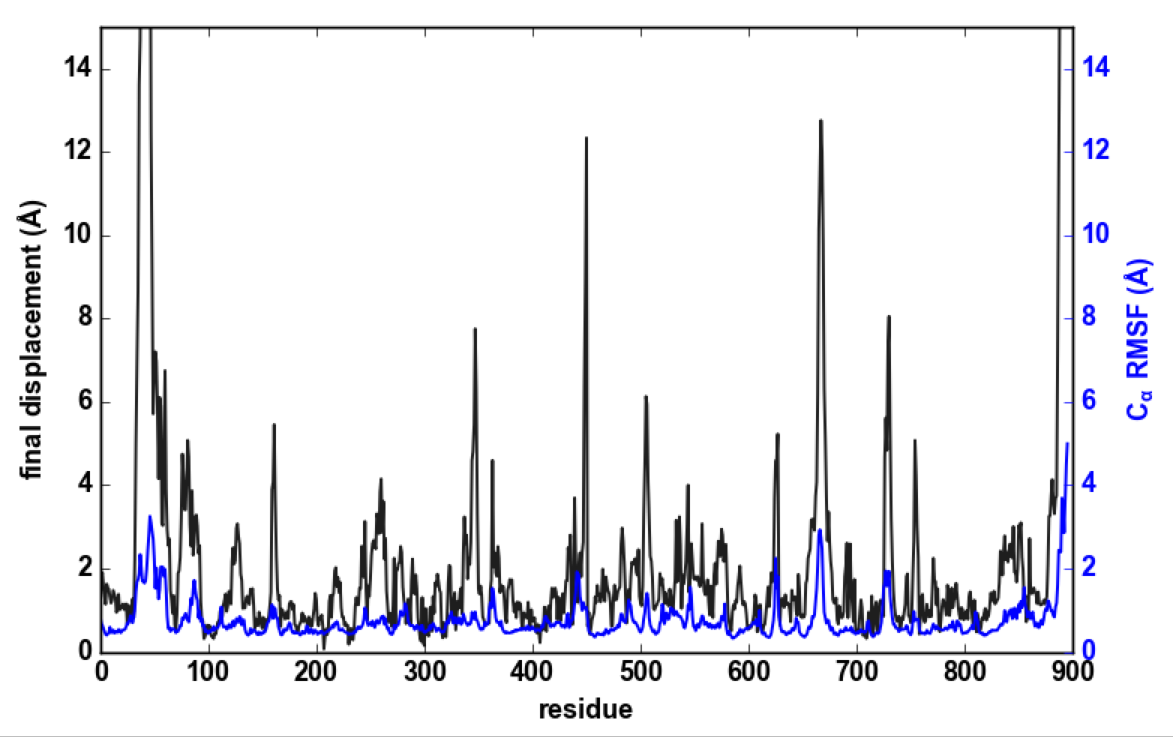

Supplement: S2 Fig — Final displacement, measured as the distance between residues after 10 ns exposure to a positive and negative transverse EEF (black). Cα RMSF per residue of a tubulin heterodimer, from the 10 to 20 ns time frame, without exposure to an EEF (blue). (TIFF) [file pone.0202141.s002.tiff]

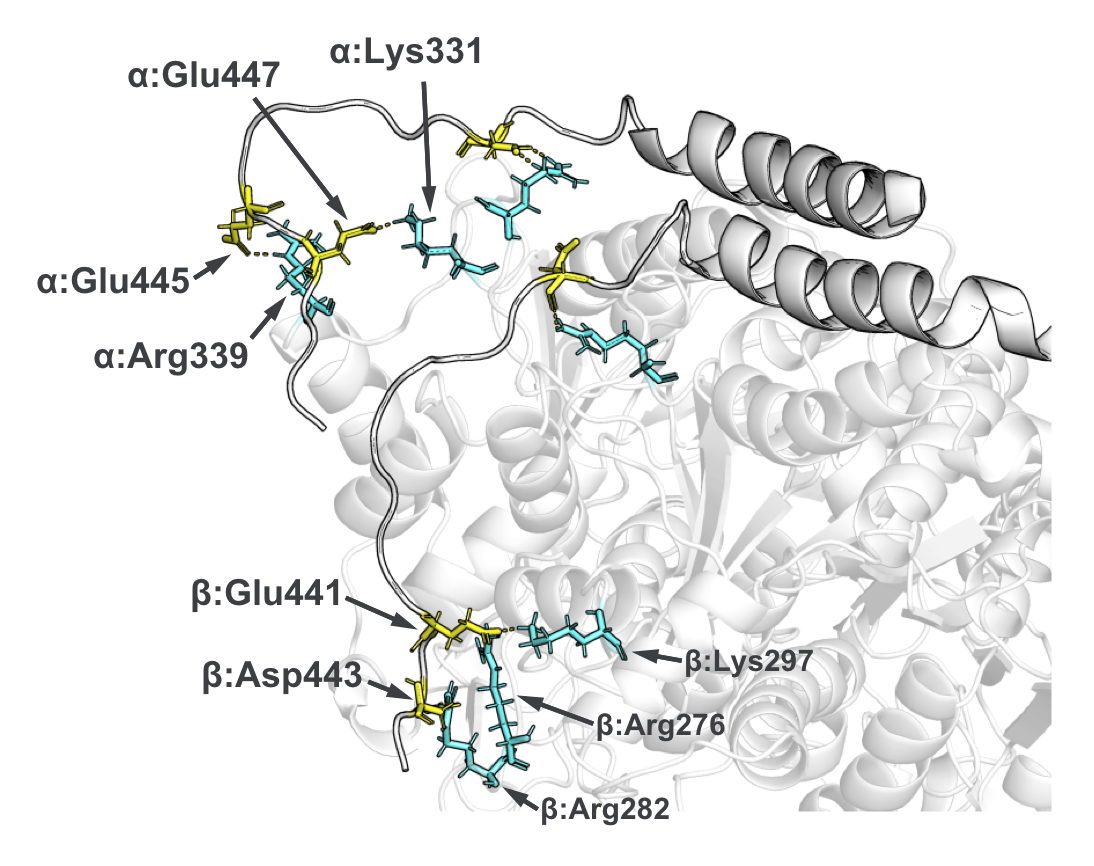

Supplement: S3 Fig — Representative salt bridges experienced by the C-termini when in a contracted state (lying along the dimer’s surface). A distance cutoff of 3.2 Å and the positions of the initial frame, after equilibration, were used to identify possible salt bridges. (TIFF) [file pone.0202141.s003.tiff]

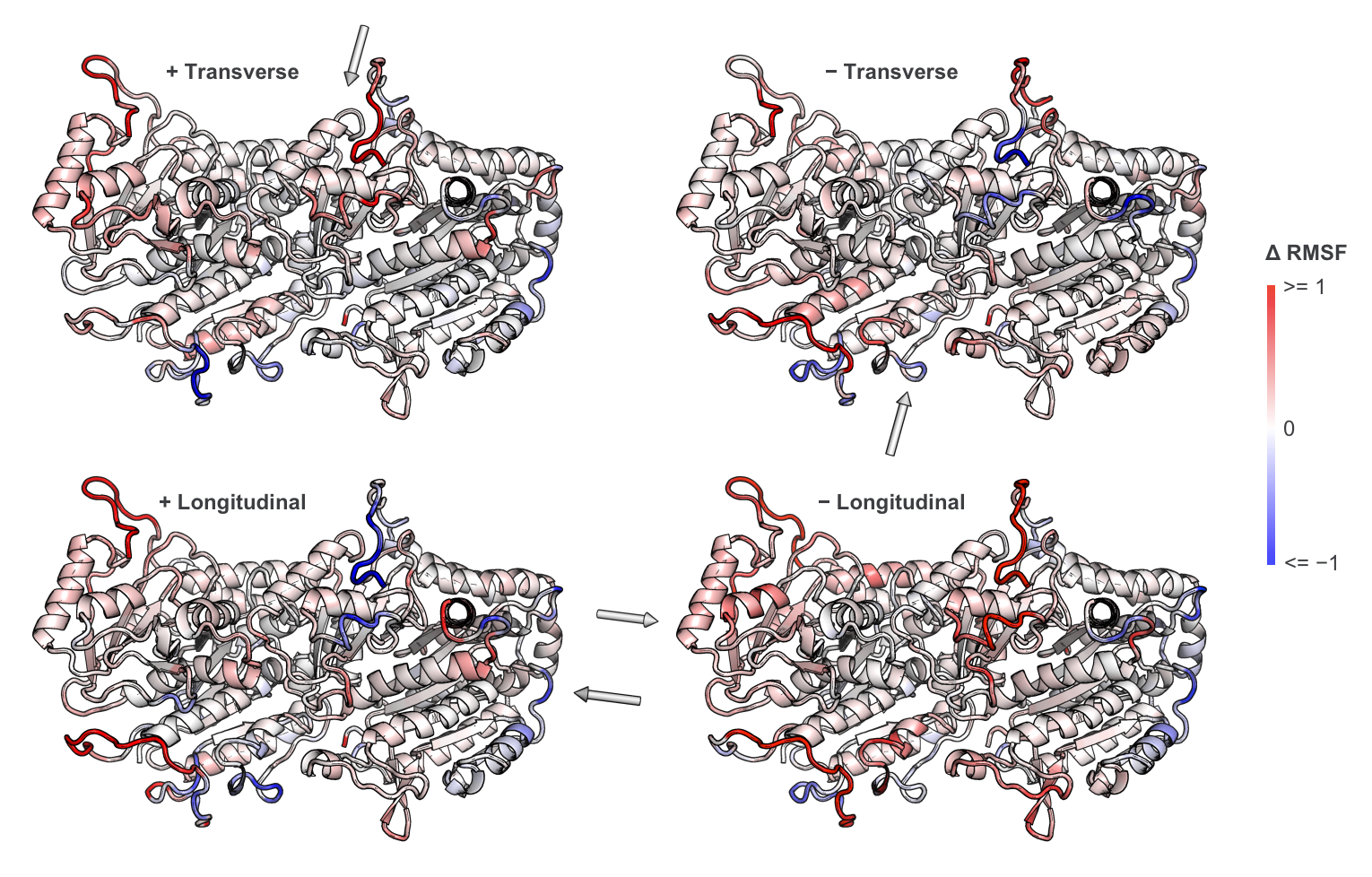

Supplement: S4 Fig — Same as Fig 4 but with ΔRMSF of each residue mapped onto the structure. Red and blue indicate an increase and decrease in RMSF, respectively. Field directions are indicated with arrows. (TIFF) [file pone.0202141.s004.tiff]

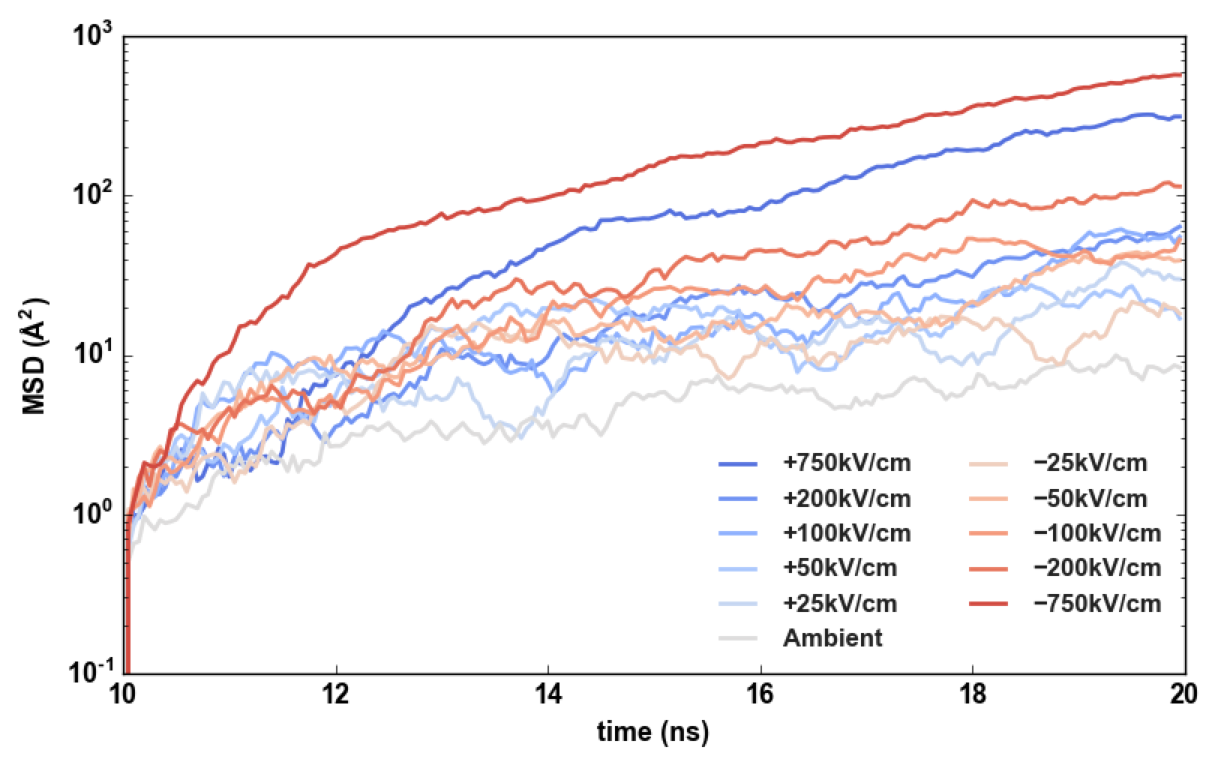

Supplement: S5 Fig — Mean standard displacement (Å2) of tubulin from the start to the end of the application of a transverse EEF. (TIFF) [file pone.0202141.s005.tiff]
